# A Popular Treatise on the Skin and Hair

**Published:** 1854-11

**Authors:** 


ART. V.—*Healthy Skin: A popular Treatise on the Skin and Hair, their Preservation and Management.* By ERASMUS WILSON, F. R. S.; Author of a Treatise on Diseases of the Skin; a System of Human Anatomy, etc., etc. Second American from the fourth and revised London edition. With illustrations. Philadelphia: Blanchard & Lea. 1854. pp. 291, 12mo.

When we first glanced at the title on the cover of this little volume we supposed it was another monograph on cutaneous diseases, but a slight examination of its contents shows it to be, what its title imports, a popular treatise on physiology and hygiene, as connected with the skin. The former subject, we must confess, has no great attractions for us. We have ever found it dull, and we felt no particular impatience to see what Mr. WILSON had contributed to its literature. But our disappointment was of an agreeable kind on finding the treatise to be really a philosophical essay, and not a mere technical account of prurigos, lichens, tetters, *et id omne genus*. Not that we undervalue the importance of works on cutaneous diseases; we admit their worth and necessity; but just at this time we were feeling the want of a treatise on the skin like the one before us—one devoted not to the treatment, but to the prevention of its diseases.

This work abounds in curious and instructive details, and most of Mr. Wilson's readers will be surprised at some of the strange phenomena pertaining to the exterior of the human body, and perhaps be ready to exclaim with Hamlet,

> "There are more things in heaven and earth, Horatio,
> Than are dreamt of in your philosophy."

Most of them are probably acquainted with the habits of the *itch insect*, which, burrowing in the skin, excites the cutaneous affection always associated in the mind of the great English lexicographer with Scotland; but here is the history of another denizen of the skin discovered by our author and delineated in his work before us. He says:—

"A few years ago, there were discovered by a German physician, Dr. Simon, in the unctous matter which collects within the oil-tubes, certain minute animals, of which he published an account in the June number of a German periodical, *Muller's Archives*, for 1842. Having read Dr. Simon's account, I set myself to work to seek for the animals in question, and before the end of June had seen some hundreds of specimens, and become so far interested in the subject, that I pursued it with almost exclusive attention for six months. In the course of this investigation, I examined many thousands of the animals, and arrived at some novel conclusions with regard to them. I found that Dr. Simon's description and figures were imperfect; that he had overlooked several points of entomological importance in the structure of the animal; as, for example, the head: and that he had left one vein of the mine, namely, the progressive development of the young from the growth and hatching of the egg, to maturity, entirely unopened. Under these circumstances, I was enabled, in the month of December, 1842, to communicate to the Royal Society a paper, since published in the *Philosophical Transactions*, containing many original observations, and upwards of forty figures of the animal. In this paper, I found myself under the necessity of changing the name given to the animalcule by Dr. Simon, as being founded on a wrong view of its structure and zoological position. As a temporary appellation, I termed it entozoon folliculorum; but, upon more mature consideration, and in reference to its habitat and food, I have thought the name *steatozoon folliculorum*, that is, the 'animal of the oily product of the skin,' more appropriate and correct.

"The animalcule of the skin is found in the oil-tubes whenever there exists any disposition to the unnatural accumulation of their contents; it is found in numbers, varying from one or two, to twenty, in the substance of the little grub-like cylinder which is squeezed out by the pressure of the fingers, and this in an apparently perfect state of health of the skin, or, more correctly, without any appearance of disorder, for the skin cannot be said to be in perfect health when its functions are performed in a torpid manner. Now, as in the majority of mankind, and certainly in all the inhabitants of cities and large towns, the skin is more or less torpid in its fucntions, so the presence of this animal in the skin is the rule; its absence, the exception. I have found it at all ages, from youth to old age, more numerously, it is true, at the latter than the former period, and in great and remarkable numbers during sickness. Under these circumstances, I see no other conclusion open than to assume that it performs some beneficent purpose in the economy of the skin; that purpose being, according to my belief, the disintegration of the over-distended cells, the impression of a new condition on the contents of the cells, and the stimulation of the tubes to perform their office more efficiently. In corroboration of this view is the fact, that these little creatures increase in numbers when the vital powers decline, so that, when the energies of the system are reduced by disease, and when the skin, participating in that reduction, is unable alone to fulfil its functions correctly, these little beings are produced to aid it in its work.

"The steatozon folliculorum is extremely minute, and quite undistinguishable by the naked eye, the longest I have seen being but little more than a quarter of a line in length; that is, forty-five, placed end to end, would measure only one inch. In form and shape, in the perfect state, they are very like caterpillars, and have a distinct head with feelers, a chest with four pairs of legs, and a long tail. The whole body is so transparent, that its interior may be easily seen, and the animal always occupies the same position in the oil-tube, the head being directed inwards, and the tail towards the aperture of the tube, as though it had crept into that situation from without. In some persons, these singular creatures are larger than in others, and in some than in other parts of the face. So much is this the case, that an eminent naturalist, to whom I showed figures of their varieties, considered the difference between the two, not merely in length, but also in shape, to amount to a specific character. If this be the fact, we may look, I imagine, for the shorter variety on the alpine ridges of the nose or eyebrows, and the longer kind in the glades and valleys of the nose and cheek. But the real cause of the difference in length I believe to be a relative diversity in the caliber of the oil-tubes in different situations, associated with a various degree of density and nutritive property of the oily matter. In the same group, also, we find eggs, embryonic forms, and young, all mingled together in confusion. In pursuing this inquiry into the animal world, I have examined them in the dog, and have found them more recently in the horse. In both these animals, the steatozoa are relatively larger than in man, and, at the same time, more tapering and slender."

We do not cite this passage as a specimen of Mr. Wilson's philosophy, with which we should be obliged to quarrel a good deal if we were going into a critical examination of his views, but we give it as an example of the curious phenomena connected with the economy of our bodies. It would be a bungling contrivance, it appears to us, to load the skin with such epizoa that it might be enabled to perform its healthy functions. Maggots in the oil-tubes of the skin, to rid them of their inspissated contents! Healthy function stimulated to greater efficiency, by the presence of animalculæ! But we pass by the theories of our author to notice some of the other interesting facts detailed in his treatise. Many instances of sudden change in the color of the hair are on record. Mr. Wilson has collected a number of these cases which we here extract:

"A lady of some literary eminence, to whom I related the foregoing instance of sudden blanching of the hair, informed me that an aunt of her own had become gray in a few days, in consequence of the shock occasioned to her nervous system, by finding, on waking in the morning, a beloved sister lying dead by her side. Mary, Queen of Scots, and Marie Antoinette both became gray in a short period, from grief. Sir Thomas Moore, we are told, turned gray during the night preceding his execution. According to Borellus, two gentlemen, the one a native of Languedoc, the other a Spaniard, were so violently affected, the first, by the announcement of his condemnation to death, the latter, by the bare thought of having incurred a serious punishment, that both became blanched in the course of a single night. The gravity with which Daniel Turner relates the following case, which he attributes to Schenkius, is amusing: "Don Diego Osorius, a Spaniard of a noble family, being in love with a young lady of the court, had prevailed with her for a private conference, under the shady boughs of a tree, within the garden of the King of Spain, but by the unfortunate barking of a little dog, their privacy was betrayed, the young gentleman seized by some of the king's guard and imprisoned; it was capital to be found in that place, and therefore he was condemned to die. He was so terrified at the hearing of his sentence, that one and the same night saw the same person young and old, being turned gray as in those stricken in years. The jailor, moved at the sight, related the incident to King Ferdinand, as a prodigy, who thereupon pardoned him, saying, he had been sufficiently punished for his fault.' And again, this, from the same author: A young nobleman 'was cast into prison, and on the morrow after, ordered to lose his head; he passed the night in such fearful apprehensions of death, that, the next day, Cæsar sitting on the tribunal, he appeared so unlike himself, that he was known to none that were present, no, not to Cæsar himself; the comeliness and beauty of his face being vanished, his countenance like a dead man's, his hair and beard turned gray, and in all respects so changed, that the emperor at first suspected some counterfeit was substituted in his room. He caused him, therefore, to be examined if he were the same, and trial to be made if his hair and beard were not thus changed by art; but finding nothing counterfeit, astonished at the countenance and strange visage of the man, he was moved to pity, and mercifully gave him pardon for the crime he committed.' And he follows this case with another, both being intended to illustrate the force of imagination. 'Somewhat like this,' says he, 'is that relation of Esquire Boyle's, who tells us, that when he was in the county of Cork, in Ireland, there was an Irish captain, who coming to deliver himself up to my Lord Broghil, commander of the English forces in those parts, according to a pardon proclaimed to those Irish that were willing to surrender themselves and lay down their arms; he was casually met with some of his followers, by a party of English, and intercepted, the governor being then absent. Upon which the poor captain was so apprehensive that he should be put to death, before my lord's return, that the very fear and anxiety of his mind quickly changed the color of his hair, in a peculiar manner, not uniformly, but interspersedly among some of his locks, which were perfectly turned white, the rest of them retaining their wonted reddish color.'

"Dr. Cassan records the case of a woman, thirty years of age, who, on being summoned before the Chamber of Peers to give evidence upon the trial of Lovel, underwent so powerful a revulsion, that in the course of one night the hair was completely blanched, and a furfuraceous eruption appeared all over her head, upon her chest, and upon her back. Henry of Navarre, on hearing that the edict of Nemours was conceded, was so exceedingly grieved, that in the course of a few hours a part of one of his mustachios whitened. In one person, some of the eyelashes became blanched from mental agitation. The writer of the article, Zoology, in the *Encyclopædia Metropolitana*, 'has known one instance of a banker whose hair became gray in the course of three days, when under much anxiety during the great panic of 1825; and also another gentleman, who, at his marriage, when about forty years old, had a dark head of hair, but on his return from his wedding trip, had become so completely snow-white, even to his eyebrows, that his friends almost doubted his identity.' Moreau narrates, that he once knew an aged man, for whom, snow-white hair and a countenance deeply marked by the furrows of care inspired the respect which we owe to age and misfortune. 'My hair,' says he, 'was as thou seest it now, long before the latter season of my life. More energetic in their effects than assiduous toil and lingering years, grief and despair, at the loss of a wife most tenderly loved, whitened my locks in a single night. I was not thirty years of age. Judge, then, the force of my sufferings; I still bear them in frightful remembrance.'

"I am little disposed to speculate on the *modus operandi* of this change of color of the hair, but am content, for the present, to give a fitting place to the fact as it stands. The phenomenon may be the result of electrical action; it may be the consequence of a chemical alteration wrought in the very blood itself; or it may be a conversion for which the tissue of the hair is chiefly responsible. In any case, the following explanation, offered by an eminent French chemist, Vanquelin, I should feel inclined to discard, as partaking too largely of the coarser opetations of the laboratory. 'We must suppose,' says the author in question, 'to explain the sudden change of the hair, that at the critical moment when nature is in revolution, and when, consequently, the natural functions are suspended or changed in nature, that an agent is developed in the animal economy, and, passing into the hair, decomposes the coloring matter. This agent must be an acid.'

"The rapid blanching of the hair derives an important illustration from the animal kingdom. Several of the animals which inhabit the polar regions are known to become white during the winter season, and among the most remarkable of these is the lemming. Sir John Ross remarks that, finding the lemming, like the polar hares which had been tamed and kept in confinement, preserve its usual color during the winter, he placed one in the open air, on the first of February, when the thermometer stood at 30° below zero. The next morning, the fur of the cheeks, and a spot upon each shoulder, had become perfectly white. On the following day the hinder part of the body, and the flanks were of a dirty white hue, and at the end of a week, the animal was entirely white, with the exception of a saddle-shaped patch on the middle of the back. No other change ensued, although the poor animal was kept exposed to the cold until it perished. When the skin was examined, the white hairs were found to be much longer than those of the unchanged patch, the blanching being confined to that portion which exceeded in length the natural hairs. So that, when the white ends were cut off, the animal appeared to have regained, with very little alteration, its summer coat, and without any reduction in the length of its fur."

Some chapters on diet, clothing, exercise, and ablutions, as affecting the health of the skin, follow the description of the structure and function of the skin and hair, and contain much instructive matter. We must indulge our readers in an extract from the latter, and so close our notice of the work, which, having read it ourselves with much pleasure, we can conscientiously recommend to our brethren.

"And now, dear reader, having determined to wash your face, how will you set about it; there are many wrong ways of effecting so simple a purpose; there is but one right way; I will tell it you. Fill your basin about two-thirds full with fresh water; dip your face in the water, and then your hands. Soap the hands well, and pass the soaped hands with gentle friction over the whole face. Having performed this part of the operation thoroughly, dip the face in the water a second time, and rinse it completely; you may add very much to the luxury of the latter part of the process by having a second basin ready with fresh water to perform a final rinsing. And now you will say: 'what are the wrong ways of washing the face? Why, the wrong ways are using the towel, the sponge, or flannel, as the means of conveying and applying the soap to the face, and omitting the rinsing at the conclusion. If you reflect, you will see at once that the hands are the softest, the smoothest, and the most perfect means of carrying the soap, and employing that amount of friction to the surface with the soap, which is necessary to remove the old and dirty scarf, and bring out the new and clean one from below. Moreover, the hand is a sentient rubber, a rubber endowed with mind; it knows when and where to rub hard, where softly, where to bend here or there, into the little hollows and crevices where dust is apt to congregate; or where to find little ugly clusters of black-nosed grubs, the which are rubbed out and off, and dissolved by soap and friction. In a word, the hand enables you to combine efficient friction of the skin with complete ablution, whereas in every other way ablution must be imperfect. Then, as regards drying the face, a moderately soft and thick towel should be used; a very rough towel is not desirable, nor one of thin texture. This is a point which may be safely left to your own taste and feelings. The question of friction during the drying is of more consequence, and this is a reason why the towel should be moderately soft, that you may employ friction and regulate the amount. With a very rough towel it is impossible to use friction, for its tenderest pressure may be enough to excoriate the skin; and a very soft towel is equally open to objection from its inadequacy to fulfil the obligation of friction during the process of drying. In washing the face you have three objects to fulfil; to remove the dirt, to give freshness, and to give tone and vigor to the skin.

"Such is ablution when intended for the purpose of cleanliness, but it must be in the experience of every one, that other effects spring from its use; that nothing is more refreshing than a thorough ablution; that, in point of fact, to those who conduct the operation properly, and with a due attention to temperature, nothing can be more luxurious, nothing restore the energies more surely and agreeably, after hours of toil or exertion; and not those of the body alone, but also, those of the mind. The poet Thomson, in his poem on the Seasons, exclaims:—

> "Even from the body's purity, the mind
> Receives a secret sympathetic aid."

"And Homer, speaking of a person just returning from the bath, and annointed with fragrant oils, says, that he appeared taller and larger than before, and was grown something like the immortals. In relation to health, the common term which we hear applied to the effects of a thorough ablution is 'bracing;' in professional language we speak of them as being 'tonic;' and in truth there exists no better means of restoring the 'tone' of the system than the judicious employment of water; and this leads me to the modes in which water may be used with the best prospect of benefit to the health.

"It must not be supposed, that because water is a good and excellent tonic, that our health would be better for being thrown into a fever by it, or even drowned in it, any more than that a parallel argument would be tenable with regard to food, clothing, or exercise. I know very well that equilibrium is not suited to the times; that there exists among mankind, in medicine as in politics and religion, a certain thirsty 'go-ahead' principle which is absolutely insatiable. I do not say that this character is the peculiar attribute of the present age, for the history of nations proves it to have existed at all periods af the world. There is no philosopher's stone of health any more than for commuting the grosser into the precious metals. But every one who desires it, has the elements of an equally valuable 'arcanum' in conforming to a correct practice of diet, clothing, exercise, and ablution. .

"The simplest mode of applying water to the skin, and that by which the smallest extent of surface is exposed, conditions of much importance to the weakly and delicate, is by means of the *wetted sponge*. In this mode, the water may have any temperature that is agreeable to the sensations, a part only of the body is exposed at a time, and as soon as that part has been *briskly* sponged, and as *briskly* wiped dry, it may be again covered by the dress. The whole body may in this way be speedily subjected to the influence of water, and to the no less useful friction which succeeds it in the operation of drying. An invalid rising from a bed of sickness would adopt this remedy by degrees, beginning first with the arms, then proceeding to the chest, and then, gradually, to the whole body. He would use warm water in the first instance, but if the season were summer, would be speedily able to proceed to cold. A person of weakly habit beginning a system of daily ablution for the first time, should commence in the spring or summer, and by the winter his powers of endurance will have become so well trained, that he will bear cold water without inconvenience. It must be admitted that the plan here laid down is very simple; it requires no apparatus, a sponge and a basin being the sole furniture for its use; but it is no less a valuable appliance to health. The cold chill of the sponge, which was at first disagreeable, becomes pleasant, the quick friction which ensues is agreeable, and while it stimulates the skin, gives action to the whole muscular system; and the warm glow, the thrill of health which follows, is positively delicious. I must, however, call attention more strongly to the 'glow of warmth' over the surface, as it is the test by which the benefit of the remedy is to be estimated, in this and in all other forms of ablution and bathing. I can hardly conceive a case in which the application of water, according to this method, could leave a chill behind it; but if such an occurrence take place, the individual has need of medical aid, and that should be promptly supplied. I may mention that it was the present form of ablution which was used by Sir Astley Cooper, and to which he attributed much of his unusually robust and excellent health.

"The second form of ablution by the sponge requires the aid of a large, shallow empty tub, or sponging-bath, in which the bather stands or sits, while he receives the water from a sponge squeezed over the shoulders and against his body. The same precautions, with regard to temperature, may be taken in this as in the preceding case, but the bather is necessarily more exposed, and the form of bathing is suitable only to persons in moderate health; excepting in the summer season, when it may be borne by invalids. In the early use of the sponging-bath, the bather should content himself with a single affusion from the sponge, and should then dry the body quickly. As there is more freedom for the limbs, there is more muscular action in this than the former method, and the glow is proportionally increased. Indeed, in the sponging-bath, exercise and ablution are combined, and its employment by persons of sedentary habits is highly advantageous. I know but one circumstance that could render the sponging-bath objectionable, and that is, the occurrence of palpitations. This, however, may be obviated, by relinquishing the drying of the body to an attendant, or adopting the 'wet-sponge,' and after a short time, if there be no tendency in the system to disease of the heart, the palpitations will cease.

"Of all the forms of cold bathing, the preceding is the most convenient, the most universally applicable, and in my opinion, the most conducive to health. It should be taken the instant we step out of bed, and may be shortened or prolonged to an extent commensurate with the temperature of the atmosphere, or the feelings of the bather. I am in the habit of recommending, that a spongeful of water should be given to each of the arms before the trunk is moistened; that the minimum for the trunk should be two spongefuls in front, and one over each shoulder for the back; that there should be one also for each leg. The whole process will not occupy more than one minute; at the end of the second minute the whole body may be dried, and the wollen garments put on. By that time is the winter of the bath made glorious summer. The reward follows quick upon the penance. In many persons, reaction is so rapid, that the whole body is enveloped in a cloud of vapor before the affusion is fully accomplished, and the comparison of Boerhaave, in reference to another condition of the skin, is aptly realized. If, says Boerhaave, the piercing chill of winter could be introduced into a summer assembly, the insensible perspiration being suddenly condensed, would give to each person the appearance of a heathen deity, wrapped in his own separate cloud. The instant the clothes are put on, the whole body feels a warm and refreshing glow. This is the kind of bath that truly deserves the meed of commendation awarded to cold bathing by Agathinus. 'They who desire to pass the short time of life in good health, ought often to use cold bathing, for I can scarce express in words how much benefit may be had by cold baths; for they who use them, although almost spent with old age, have a strong and compact pulse, and a florid color in their face; they are very active and strong, their appetite and digestion are vigorous, their senses are perfect and exact; and, in one word, they have all their natural actions well performed.' To which Sir John Floyer adds, the effects of cold bathing 'reach the very soul of the animal, rendering it more lively and brisk in all its operations.'"

